# A Review of the Nutritional Aspects and Composition of the Meat, Liver and Fat of Buffaloes in the Amazon

**DOI:** 10.3390/ani14111618

**Published:** 2024-05-29

**Authors:** Laurena Silva Rodrigues, Jamile Andrea Rodrigues da Silva, Welligton Conceição da Silva, Éder Bruno Rebelo da Silva, Tatiane Silva Belo, Carlos Eduardo Lima Sousa, Thomaz Cyro Guimarães de Carvalho Rodrigues, André Guimarães Maciel e Silva, José António Mestre Prates, José de Brito Lourenço-Júnior

**Affiliations:** 1 Postgraduate Program in Animal Science (PPGCAN), Institute of Veterinary Medicine, Federal University of Para (UFPA), Castanhal 68746-360, Brazil; laurena.rodrigues@ifac.edu.br (L.S.R.); eder.b.rebelo@gmail.com (É.B.R.d.S.); thomazguimaraes@yahoo.com.br (T.C.G.d.C.R.); andregms@gmail.com (A.G.M.e.S.); joselourencojr@yahoo.com.br (J.d.B.L.-J.); 2 Institute of Animal Health and Production, Federal Rural University of the Amazônia (UFRA), Belem 66077-830, Brazil; jamileandrea@yahoo.com.br; 3 Department of Veterinary Medicine, University Center of the Amazon (UNAMA), Santarem 68010-200, Brazil; tatianebelovet@gmail.com (T.S.B.); cadu34.medvet@gmail.com (C.E.L.S.); 4 Center for Interdisciplinary Research in Animal Health (CIISA), Faculty of Veterinary Medicine, University of Lisbon, 1300-477 Lisboa, Portugal; japrates@fmv.ulisboa.pt; 5 Associate Laboratory for Animal and Veterinary Science (AL4Animals), 1300-477 Lisboa, Portugal

**Keywords:** Amazon, fatty acids, minerals, vitamin

## Abstract

**Simple Summary:**

This review aims to deepen the understanding of buffalo farming in the Amazon, presenting the quality and nutritional value of buffalo meat and liver. We will explore the characteristics of nutrients and their influence on human health, with a special focus and particular emphasis in vitamins, minerals, and lipids. By investigating fatty acids and cholesterol, we seek to understand the complexity of lipids and their contribution to nutritional quality. The main objective of this study was to provide subsidies to improve buffalo farming practices, promote a healthier human diet and contribute to environmental sustainability in the Amazon region.

**Abstract:**

Thus, this review aims to deepen the understanding of buffalo farming in the Amazon, presenting the quality and nutritional value of buffalo meat and liver. This information serves as a subsidy to improve practices related to the breeding system, nutrition, health and sustainability associated with aquatic buffaloes. For this, a review of the databases was carried out using the descriptors “nutritional value of buffalo meat”, “nutritional value of buffalo liver” and “buffalo breeding in the Amazon”. Thus, the consumption of foods derived from aquatic buffaloes has important nutritional value for human consumption. In view of this, it is possible to conclude that the nutrition of these animals is influenced by the biodiversity of the Amazon, giving unique characteristics to its products, also highlighting the importance of carrying out research that aims to value the potential use of this species and strengthen the economy of the region.

## 1. Introduction

Aquatic buffaloes, species *Bubalus bubalis*, family Bovidae and subfamily Bovinae, have Asian origins, and are found in practically all continents, such as Europe (Italy), Africa (Egypt), Asia (India, Pakistan, Thailand, China, and Vietnam) and South America (Brazil, Argentina, Venezuela, Peru, and Colombia) [1,2,3]. In Brazil, the buffalo was introduced to the island of Marajó, Pará, in 1890, where they lived in natural conditions. At the end of the 1950s, they began to be raised in managed systems, adapted to the tropical conditions of the Amazon, and demonstrated high aptitude in meat and milk production and work, in addition to excellent reproductive characteristics, without causing damage to the environment [4,5,6].

Currently, the Brazilian buffalo herd has 1,598,268 million heads, of which 73% are in the Amazon, with more than 1 million heads [7]. In Pará, most of the aquatic buffaloes are found on Marajó Island and in the Lower and Middle Amazon. Aquatic buffaloes are long-lived and docile and have high reproductive and productive efficiency, with the capacity to occupy 100% of the available territory and transform low-quality grasses into products of outstanding added value [8,9].

Buffalo meat, when compared to beef, has a lower degree of marbling, is leaner, and has more protein, greater pigmentation, and higher dry matter content. It has 40% less cholesterol, 12 times less fat, 10% more minerals, 55% fewer calories, and 11% more protein [10,11,12]. Due to its superiority in terms of nutritional value over conventional (beef) and white meat (chicken), buffalo meat is an important source for the production of processed products of exceptional quality, such as smoked sausage [13,14,15,16,17].

In fact, buffalo production represents a viable alternative for efficiently using flooded areas that would otherwise not be used by other more conventional species, such as cattle or sheep. In addition, we can use technologies for developing buffalo farming in pastures cultivated in rainfed areas, through the recovery of eroded or overgrazed pastures [18,19,20]. Still, in relation to food security, the supply and consumption of food of animal origin increasingly require an increase in the production and breeding of animals in order to meet the food demand of the population. Thus, raising buffaloes for commercial purposes and for human consumption can be a viable alternative to supply human consumption, especially in regions with low agricultural potential [21,22].

Based on this information, the formulation of a literature review on the different nutritional aspects, as well as the composition of the liver and meat of buffalo in the Amazon, helps to fill the gaps in our knowledge and maximizes the chance of understanding the characteristics of the meat consumed. In addition, the growing buffalo population in the Amazon contributes to the search for knowledge about the nutritional aspects of animal products from this species. Thus, this review aims to deepen the understanding of buffalo farming in the Amazon, presenting the quality and nutritional value of buffalo meat and liver.

## 2. Materials and Methods

We searched the databases of Scopus, PubMed and Web of Science. The following descriptors were used: “nutritional value of buffalo meat and liver”, “nutrition of aquatic buffaloes” and “feed of aquatic buffaloes”. The articles were selected according to the inclusion criteria, which were full texts, in English, Portuguese, Spanish and Italian, on human food and nutrition and the chemical composition of meat and liver of Amazonian aquatic buffaloes, and all articles that did not fit the objective proposed by this study were excluded. To search for the articles, the geographical limits for the Amazon region were considered; therefore, 110 publications are cited in this review.

## 3. Contextualization

### 3.1. Buffalo Farming in the Amazon

The buffalo herd was introduced to the Brazilian territory in 1890 on Marajó Island, Pará, located at the mouth of the Amazon River [23,24]. These animals have adapted very well to the environmental conditions of the Amazon due to the similarities with those of their countries of origin, characterized by a predominantly tropical climate [25,26]. They remained there and developed in the conditions of the island due to the ease of adaptation to the most varied types of environment, thus reaching satisfactory zootechnical indices such as average daily gain, carcass yield and fertility index.

Water buffaloes are docile animals with a high level of hardiness, tolerance and resistance to contagious and/or parasitic diseases and an incomparable longevity. In addition, these animals are highly adaptable to different regions of the globe. In general, they are bred in wetlands, marshes and riverside areas, where they have a greater ability to move and dissipate heat, as well as feed on plants native to these areas. Their ability to swim gives them greater resistance to challenging climatic conditions, such as seasonal flooding [20]. 

Compared to other ruminants, aquatic buffaloes have some peculiarities in their capacity to use nutrients (dry matter, crude fiber, crude protein and nitrogen balance) [18]. The fiber and protein consumed by ruminants, obtained from different sources and varied compositions, have an influence the microbial that low-quality forages are better used by aquatic buffaloes [27]. 

**Figure 1:**
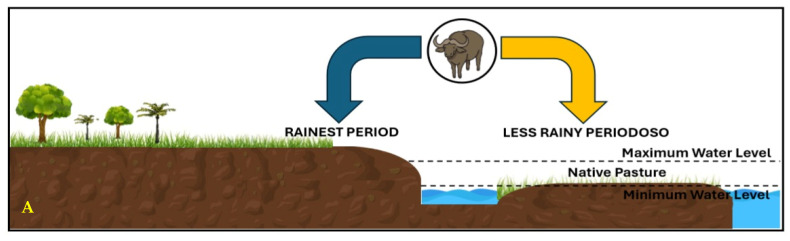

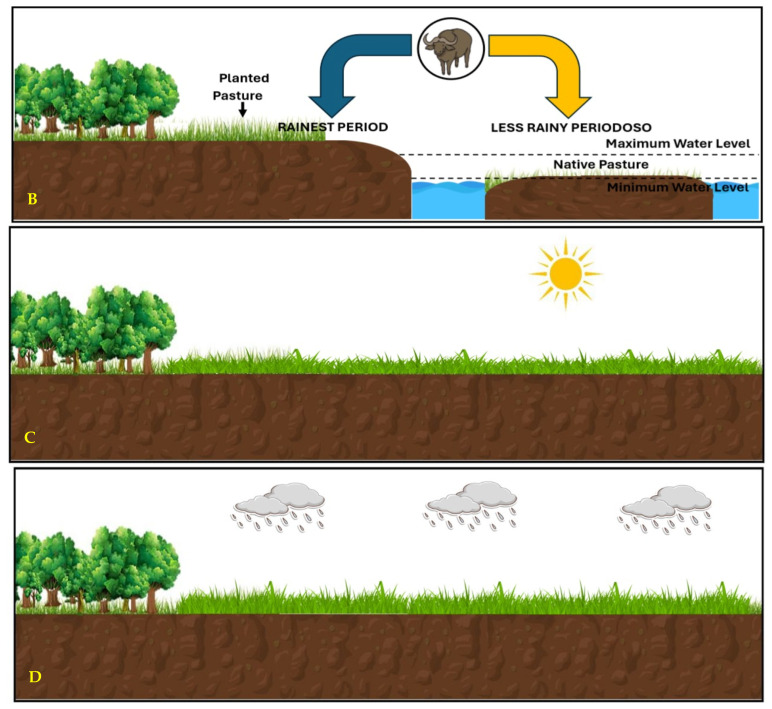
Characterization of the three ruminant farming environments in the State of Pará. (**A**). Natural grasslands. (**B**). Dryland and floodplain. (**C**). Dry land: less rainy period. (**D**). Dry land: rainy season.

In the Brazilian Amazon, buffalo farming is developed in pasture-based breeding systems, with four distinct pasture ecosystems: 1—native to flooded areas of the estuary, with an area of approximately 50 thousand km^2^, distributed on the island of Marajó, where most of the herd is concentrated. The topography is flat, with small unevenness that distinguishes two areas: the low grasslands—areas that remain flooded six months or more a year, and the high grasslands—higher areas that are not affected by floods (Figure 1A); 2—native to floodable areas located in the micro-regions of the Lower and Middle Amazon, with floodplain areas, which comprises the part periodically flooded by the flooding of the Amazon River [24,26]. Each year, when the river level rises, native pastures flood, farmers opt for two management strategies. Traditionally, they keep the animals in hanging pens called “marombas”, feeding them with floating grasses that detach from the land because of the flood, being collected in canoes. With the construction of the Cuiabá—Santarém Highway, it became common to transport animals to pastures planted on dry land (Figure 1B); 3—cultivated on dry land (Figure 1C); and 4—cultivated on dry land, in areas originally of forest confinement (Figure 1D). In the latter ecosystem, more productive forages with better nutritional value have been used in herds formed by animals with a better genetic standard [28]. Figure 1 shows the characterization of the three ruminant farming environments in the State of Pará and the map of ruminant rearing in the state of Pará.

The native pastures of the island of Marajó are very stable ecosystems that have been exploited by beef cattle in an extensive management system for more than 300 years, with reduced levels of degradation. Native pastures in floodplains, especially those in the Lower and Middle Amazon regions, have played a very important role in the context of regional cattle ranching [19].

**Figure 2:**
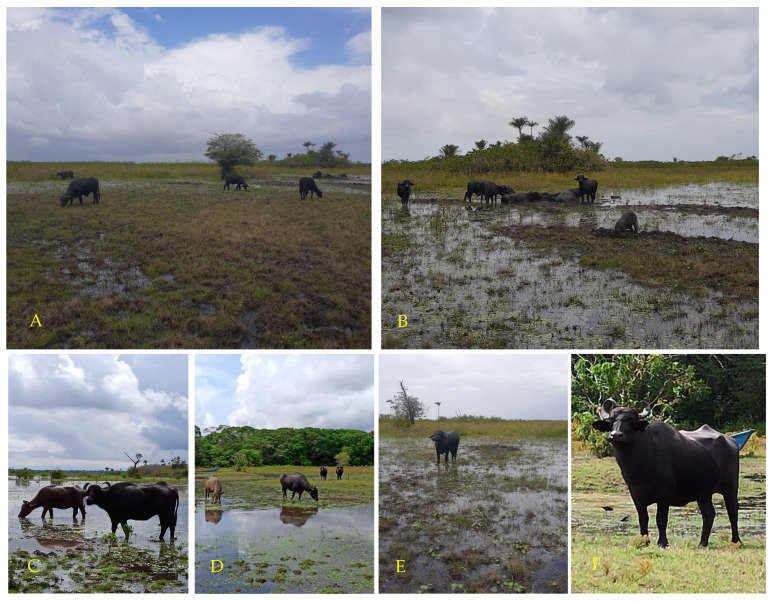
Aquatic buffaloes in flooded pastures at the end of the rainy season in Santarém, Pará, Brazil, Lower Amazon region (**A**–**F**).

The Figure 2 shows aquatic buffaloes in flooded pastures at the end of the rainy season in the Lower Amazon, Santarém, Pará.

Native floodplain pastures play a fundamental role in the development of buffalo breeding in the Brazilian Amazon, as they have a high the most significant potential for producing forage of good nutritional value. These pastures are located on the banks of the Amazon River and its tributaries, muddy water lakes and areas of its estuary. The largest extensions of these pastures are found in the micro-regions of the Lower and Middle Amazon and part of the island of Marajó [29,30]. 

Due to its excellent characteristics in milk, meat and work production, combined with its high adaptability to different Brazilian environmental conditions, the buffalo is an animal of great representativeness for the national livestock [4]. However, currently, the consumption of buffalo meat is restricted only in the North and South regions of the country. The limiting factor for the consumption and commercialization of the products produced by these animals is the lack of information on their chemical constituents [31]. 

### 3.2. Quality and Nutritional Value of Buffalo Meat and Liver

Aquatic buffaloes have high aptitude as a producer of protein of high biological value, so they can reach weights above 500 kg at 3.5 years of age in natural fields in the northern region of Brazil [32]. There is excellent sensory conformity of the meat of young aquatic buffaloes, compared to the “lean” beef of the zebu, in terms of juiciness and tenderness. It is a meat that denotes a property that grants its inclusion in the class of healthy foods because it contains high levels of protein and low contents of fat and cholesterol, when compared to beef [33].

Lean buffalo meat has around 75% water, 21 to 22% protein, 1 to 2% fat, 1% minerals and less than 1% carbohydrates [34]. Raw meat has an average of 105 kcal/100 g of energy content, which is relatively low. Higher values were found in fat: 830 kcal/100 g (Table 1 and Table 2). Male aquatic buffaloes fattened on cultivated *Brachiaria humidícola* pasture and slaughtered at two and a half years of age contained good levels of nutrients in fillet samples in terms of water (76.23%), dry matter (23.77%), protein (20.69%), fat (1.98%) and minerals (1.10%) [35,36,37].

The significant amounts of zinc, B vitamins, and cholesterol found in buffalo meat can serve as an excellent source of these nutrients for pregnant women. In addition, it has a high protein content (21.13 g/100 g), reduced amounts of lipids, saturated and monounsaturated fatty acids, and (1.37 g, 0.460 g, and 0.420 g), respectively, as well as essential nutrients for all stages of life when appropriately consumed [38,39].

It should also be noted that the quality and bioavailability of nutrients can be variable, depending on several factors, such as management, nutrition and welfare, since these are crucial when it comes to impacts on animal productivity. Thus, this is an excellent food option for the human population [40].

### 3.3. Characteristics of Nutrients and Their Relationship with Human Health

Another exciting factor is that the fatty acid profile found in buffalo meat differs from that found in cattle and pigs, which have lower levels of saturated fatty acids (SFA) and polyunsaturated fatty acids (PUFA), such as omega-3. These are positive and desirable factors for healthy human nutrition [41,42].

Nutritionally, meat is an excellent source of vitamins, minerals, essential fatty acids and amino acids. Vitamins A and B12, for example, are relevant nutrients; they are found only in foods of animal origin, the most important being meat and liver [43].

In a research study carried out in Belém do Pará, Brazil, it was observed that the group of people who had a diet including a high consumption of meat, fruits, cereals, milk and dairy products and a low consumption of processed foods exhibited positive results with a healthier life. This is because the exaggerated consumption of processed and ultra-processed foods and a deficit of fresh and minimally processed foods are risk factors for cardiovascular diseases and comorbidities [44].

### 3.4. Vitamins

Among the essential nutrients for life are vitamins, which are organic substances found in food and are indispensable for the proper functioning of the body. Thus, an adequate diet is one of the preponderant factors for the healthy growth and development of human beings [45]. Hypovitaminosis can result in growth or development deficits, as well as disorders and deficiencies related to low vitamin intake, leading to deficiencies [46,47]. 

Regardless of the environment, most living organisms are unable to synthesize vitamins anabolically, which is why they need to be included in diets; in general, they are needed in microquantities and depend on the age, sex, physiological state and physical activity of the individual. The requirements for these nutrients increase during the periods of growth, gestation and lactation, in conditions of intense work, and when there is an occurrence of certain diseases, notably infectious diseases [48].

Vitamins are classified into fat-soluble and water-soluble, according to the physiological and physicochemical properties common to them. The fat-soluble vitamins are vitamins A (retinol), D (calciferol), K (phylloquinone) and E (tocopherol), and the most common water-soluble vitamins are represented by B vitamins (B1: thiamine diphosphate; B2: riboflavin; B3: niacin; B5: pantothenic acid; B6: pyridoxal-5-phosphate; B8: biotin; B9: folate and B12: cyanocobalamin) as well as some organic acids, such as ascorbic acid (vitamin C) [49]. However, in this study, only vitamin E and β-carotene will be treated. 

Retinoic acid leads to the separation, maturation, and function of innate immune cells, which are made up of macrophages and neutrophils and spearhead immediate feedback to the invasion of pathogens by phagocytosis. Another important factor is that retinoic acid activates natural killer T-cells, which perform immunoregulatory functions through cytotoxic activity [50]. Living organisms do not synthesize vitamin A, so it needs to be supplied through food [51,52]. 

Vitamin A is also known as an anti-inflammatory vitamin as it helps maintain the body’s immune system, which is why its consumption can help improve inflammatory processes. In addition, this vitamin is associated with vision, and deficiencies can lead to blindness [53].

Vitamin E, the largest lipolytic antioxidant, is a dietary of great importance for the health of humans and animals. Its main shape is found in plasma and erythrocytes in the liver. This micronutrient has four tocopherols and four tocotrienols, designated α (alpha), β (beta), γ (gamma), and δ (delta). Tocopherols have a phytyl chain; however, tocotrienols have a similar chain, but with three double bonds at positions 3, 7 and 11. They are differentiated by the number and position of methyl groups in the aromatic ring [54], and their most relevant form is α-tocopherol, thanks to its high biological antioxidant activity detected in high concentration in immune cells, compared to other blood cells [47]. 

Vitamin E has a positive immunomodulatory impact on attenuating various viral, bacterial and allergic diseases, such as asthma, cataracts, cancer. It is apt to modulate the innate immune response to pneumonia infection and improve lung pathology [55].

### 3.5. Minerals

Minerals are substantially inorganic elements, usually metals, that account for between 4% and 5% of body mass. They are detected in the body in the form of ions, which are fundamental to a plurality of functions, such as cofactors of enzymatic reactions in acid–base balance, nerve impulses, and muscle activity and as structural constituents of organs and tissues, in addition to being present in body fluids [56,57].

Minerals are divided into three classes according to the amount in which they are present in the body. Macroelements represent the minerals required in greater quantities (>0.005% of body mass) by the body. They are represented by calcium (Ca), phosphorus (P), sulfur (S), magnesium (MG), potassium (K), and sodium (Na). See Table 3 for the main functions of macrominerals.

Microelements are the minerals identified in smaller amounts (<0.005% of the body mass), which are copper (Cu), cobalt (Co), chromium (Cr), tin (Sn), iron (Fe), fluorine (F), manganese (Mn), iodine (I), molybdenum (Mo), nickel (Ni), selenium (Se), silicon (Si), vanadium (V), and zinc (Zn). The third class of minerals is the elements found at trace tissue levels, such as arsenic (As), boron (B), cadmium (Cd), lead Pb), strontium (Sr), lithium (Li), and mercury (Hg), all of which show no known attribution in the human body [17,56].

It is important to highlight that essential micronutrients, although they constitute only 4% of body weight, perform vital activities in the body, with repercussions on animal performance. The lack of one or more mineral elements can trigger serious nutritional disorders, which can lead the animal to deficits in productive and reproductive performance, so that they have the physical conditions to express their full potential [59]. This same imbalance in nutritional status, malnutrition, leads to a deficiency in the immune system not only of the animal, but also in the human body. A person’s nutritional needs should be assessed based on age, gender, and physical activity, among other factors [60,61]. See Table 4 for the main trace minerals.

**Figure 3:**
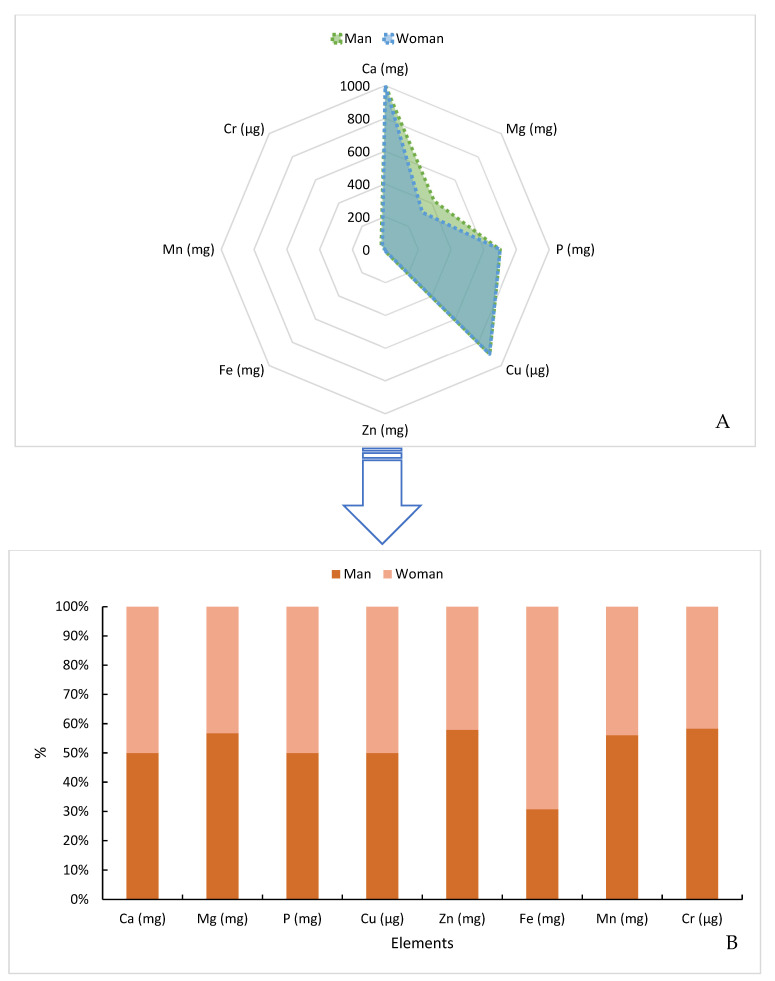
Daily values of adequate mineral intake for men and women between the ages of 19 and 50. (**A**). Raw data. (**B**). Percentage data. Note: Male (400 mg) and Female (310 mg) up to 30 years of age. Adapted from PADOVANI et al. [62].

A person’s nutritional needs should be assessed according to age, gender, physical activity, among other aspects. Thus, in Figure 3, we provide some daily values of adequate mineral intake for men and women between the ages of 19 and 50.

Rodrigues et al. [16] showed that adequate levels of minerals such as sodium (Na), potassium (K), calcium (Ca), magnesium (Mg) and phosphorus (P) are found in the liver of aquatic buffaloes raised in Marajó. These nutrients are indispensable for human nutrition, since they act by regulating the body’s water balance, nerve impulses, muscle contraction and relaxation, blood pressure, coagulation process, maintenance of bone health, secretion of hormones, formation of antibodies, and bones and teeth [63,64,65,66].

### 3.6. Lipids

The knowledge that diet and human health are closely linked has led society in general to seek out healthier, functional foods with important nutritional value for human health. In view of a more demanding consumer profile, it is essential to identify protein sources with a previously known lipid composition [67,68].

Lipids are biological compounds that, regardless of the disparities between them, show common attributes, such as low solubility in water and other polar solvents, and high solubility in nonpolar solvents. Commonly recognized as fats, lipids have the hydrophobic physical nature of molecules, which is not a biological drawback. They are essential to determine an interface between the intracellular and extracellular medium and are hydrophilic [49,69].

In nutrition, nutrients are important and generally classified into water, protein, carbohydrates, minerals, vitamins and lipids. Water is important for health and life since its absence causes loss of functions and even death of the individual. However, the most misunderstood group is lipids [70]. This fact is true for two reasons: lipids are mere energy providers and all fatty acids are equal and without individual benefits [71]. However, lipids are very important sources of energy, accounting for more than 40% of a newborn’s energy supply from breast milk. In addition, they act in the transport of fat-soluble vitamins and as hormonal precursors, while carbohydrates act as a primary source of energy and proteins act as builders and are involved in the synthesis and maintenance of hormones and muscles, respectively [72].

### 3.7. Fatty Acids Composition

Compared to cattle meat, buffalo meat and fat are rich in branched-chain fatty acids, monounsaturated fatty acids, oleic acid, and polyunsaturated fatty acids like linoleic acid [33]. It should be noted that buffalo fat contains nutrients considered nutraceuticals, used in human health, due to their anticarcinogenic and cardioprotective function [37,73].

Also, when consuming buffalo meat and fat, N-6 and N-3 fatty acids can be found, which are polyunsaturated fatty acids indispensable to human health and need to be ingested through the diet or supplemented. In animal products, eicosapentaenoic acid (EPA), docosahexaenoic acid (DHA), and docosapentaenoic acid (DPA) can be found, which are involved in the synthesis of lipoxins and prostaglandins and have an anti-inflammatory function [74,75,76].

Fatty acids are essential nutrients for life as they are used by organisms as a source of energy. They are involved in metabolic regulation and immune modulation [77,78,79]. Saturated fatty acids (SFAs) have only single bonds in the carbon atom and the maximum number of hydrogens bonded to the carbon. When unsaturation occurs, one or more hydrogen atoms are dispensed, and a double bond is formed between two carbons. If there is only one double bond between the carbon atoms, they are called monounsaturated fatty acids (MUFAs), and if there are two or more double bonds, they are called polyunsaturated fatty acids (PUFAs) [80].

Fatty acids are of paramount importance for the survival and proper performance of vertebrate metabolism. Some fatty acids are not synthesized by the body; they are so-called essential fatty acids, which must be supplied through the diet, and include linoleic acid (18:2 ῳ-6) and linolenic acid (18:3 ῳ-3) [81,82]. Polyunsaturated fatty acids have been linked to the prevention and treatment of many chronic diseases, such as neurological, cancerous, inflammatory diseases; obesity; and diabetes mellitus [59,83,84,85].

However, the anticholesteremic characteristics of monounsaturated fatty acids are possibly due to oleic acid (18:1 cis-9), since monounsaturated fatty acids such as elaidic acid (18:1 trans-9), palmitoleic acid (16:1 cis-9) and myristoleuc acid (14:1 cis-9) do not share the same properties [86,87]. The fat level in meat induces its fatty acid profile, regardless of species, breed or diet. The SFA and MUFA content increases more rapidly with the increase in fat content, as a result of a decrease in the proportion of PUFAs and, consequently, in the PUFA/SFA ratio. Ruminant fat is characterized by having high SFA content and a low PUFA/SFA ratio relative to monogastric animal fat [88,89].

The search for alternative and complementary sources of omega-3 polyunsaturated fatty acids has become indispensable for changing the ratio between the sum of omega-6/omega-3 acids (n-6/n-3) consumed. The amounts of fatty acids and the ratios between the fatty acids of the n-6 and n-3 families, currently ingested by humans, are difficult to examine since they are subordinated to the physiology, availability or variety of food and the diet of each individual. We have not yet accurately determined the minimum levels of fatty acid intake of the n-3 and n-6 series that meet the human requirements of these nutrients; however, it is necessary to consume a balanced diet with n-3 and n-6 fatty acids in the diet [90,91].

### 3.8. Cholesterol

Cholesterol is a fundamental compound of all cell membranes. It is the biosynthetic indicator of bile acids, vitamin D, and steroid hormones synthesized by the adrenal glands and gonads (testes and ovaries) and has a very important function in nervous tissues [59]. Cholesterol is a lipid that is mainly produced endogenously, in addition to being transported by the blood to all the cells of the body. It is obtained through food. However, high blood cholesterol levels increase fat retention in the arteries’ walls, consequently increasing cardiovascular disease risk [92,93].

Lipids are conducted within body fluids in the form of lipoprotein particles (triglycerides, phospholipids, cholesterol) and another protein (apolipoproteins), which are identified according to the size and density [94] of their particles, which are a function of the relative content of cholesterol and triglycerides, or by the type of apolipoprotein.

The reduction in the consumption of foods rich in saturated fatty acids and cholesterol is a key factor in the prevention of obesity, hypercholesterolemia, cardiovascular diseases, diabetes, and in reducing the risk of the onset of some types of cancer [95,96]. Given this, there is no reason to cease or drastically reduce meat consumption since it has primordial nutritional components essential to health [97,98] and can even be considered a functional food.

Trans fatty acids are not essential and do not attribute any known benefit to human health [99]; on the contrary, they favor the increase in LDL cholesterol levels and originate in the processing of many foods [49,69,100]. Trans fatty acids have been shown to increase the risk of cardiovascular disease more than any other macronutrient [19,101] and substantially increase risk, even with low levels of consumption (1 to 3% of total energy intake), so the World Health Organization recommended in 2003 that trans-fat consumption should be limited to less than 1% of total energy intake [32,59,102].

### 3.9. Nutritional Value of Lipids

Much more than judging the quantity of lipids in food, one should ascertain the quality of lipids in the diet of Western populations [91]. The quality of lipids is the main factor in reducing the risk of cardiovascular disease and other diseases that can arise in the human body due to a high consumption of foods with low-quality fatty acids [103,104].

A range of studies reveal beneficial effects of fatty acids on human health and specific results in fighting, controlling, and/or preventing many chronic diseases [48,95]. For these and other reasons, it is essential to establish nutritional criteria that can help assess the nutritional quality of food.

Genuinely, ruminant diets include lipids rich in unsaturated fatty acids, emphasizing that forages and oilseeds contain high portions of polyunsaturated fatty acids, especially linoleic and linolenic fatty acids, which are considered essential fatty acids [100,105]. 

Generally, meat from pasture-raised animals has a higher content of n-3 fatty acids than meat from animals fed concentrates [106]. The Western diet is characterized by a disharmony between n-6 PUFA and n-3 PUFA, with an n-6/n-3 ratio much higher than recommended [83,84,107,108]. The disproportion of the n-6/n3 ratio represents a risk factor in the development of some types of cancer and cardiovascular diseases. The increased consumption of foods that provide n-3 fatty acids reduces the incidence of these diseases [109,110].

### 3.10. Future Prospects

Future prospects in relation to the nutritional parameters and elements of the meat, liver and adipose tissue of aquatic buffalo have provided insights for further research and applications. In this same scenario, the growing evolution of consumer preferences in choosing healthier and more sustainable foods has provided opportunities for niche markets and products derived from buffaloes raised in the Amazon region.

Likewise, with the growing evolution in research and technology, a probable understanding of the nutritional composition of aquatic buffalo products and their possible health consequences is likely to improve. In addition, other studies can focus on recognizing specific bioactive complexes present in these products and proving their physiological effects on population health. As a result, a dedication to improving production systems and animal husbandry practices can help to strengthen the nutritional quality and overall sustainability of food derived from water buffaloes, making them important contributors to the future of varied and nutritious food supplies.

## 4. Conclusions

Based on the analysis of the nutritional values and meat, liver and fat composition of aquatic buffaloes raised in the Amazon region, it can be concluded that

1. These products have distinct characteristics compared to those from other regions.
2. The rich Amazonian biodiversity directly influences the diet of aquatic buffaloes, giving unique properties to their products. Their meat, for example, reveals a differentiated nutritional value, enriched by specific elements of the local flora.
3. Similarly, the liver exhibits remarkable concentrations of essential nutrients, while the fat exhibits specific fatty acid profiles that may benefit human health.

Thus, this study on recognizing the varied aspects of these products provides opportunities for marketing and the progression of diversified food products, adding to local economic value and promoting cultural appreciation and the natural resources of the Amazon region. Therefore, by highlighting the nutritional advantages of these products, this study can help promote healthier and more sustainable diets for the local and global population.

## Figures and Tables

**Table 1:** Nutritional value per 100 g of buffalo meat compared to cattle meat.

Nutrients	Buffalo	Cattle
Proteins	21.13 g	19.23 g
Isoleucine	5.0 g	4.8 g
Leucine	8.1 g	8.1 g
Lysine	8.4 g	8.9 g
Methionine + Cystine	3.9 g	4.0 g
Phenylalanine + Tyrosine	6.7 g	8.0 g
Threonine	3.8 g	4.6 g
Tryptophan	1.0 g	1.1 g
Valine	4.5 g	5.0 g
Myristic acid	15.3 mg	9.9 mg
Palmitic acid	189.3 mg	143.9 mg
Stearic acid	173.7 mg	117.7 mg
Oleic acid	253.0 mg	267.3 mg
Total SFA	383.0 mg	275.7 mg
Total PUFA	255.7 mg	267.9 mg
Total MUFA	182.0 mg	175.0 mg
Cholesterol	41.3 mg	60–90 mg
Thiamine	0.045 mg	0.15 mg
Riboflavin	1.7 mg	0.26 mg
Niacin	20 mg	6.30 mg
Pyridoxine	0.253 mg	0.30 mg
Cobalamin	2.131 mg	1.00 mg
Iron	2.56 mg	1.2 mg
Zinc	4.0 mg	3.2 mg
Magnesium	24.2 mg	25.7 mg
Calcium	5.1 mg	7.4 mg
Phosphorus	181.1 mg	109.4 mg
Potassium	290.3 mg	312.4 mg
Sodium	74.6 mg	61.9 mg

Note: SFA = Saturated Fatty Acids; PUFA = Polyunsaturated Fatty Acids; MUFA = Monounsaturated Fatty Acids. Adapted Newton et al. [26]; Nikolau et al. [27]; Ponnampalam et al. [32]; Failla et al. [33]; Mali et al. [34]; Baran et al. [35]; Rébak et al. [36].

**Table 2:** Nutritional value of buffalo liver compared to beef liver.

Nutrients	Buffalo	Cattle
Proteins	21.4 g	20.7 g
Total lipids	40.72 mg	5.4 g
Total cholesterol	2.57 mg	393 mg
Lauric acid	0.15 mg	3.25 mg
Myristic acid	0.34 mg	0.005 mg
Palmitic acid	17.66 mg	24.4 mg
Stearic acid	25.24 mg	18.8 mg
Oleic acid	18.15 mg	6.77 mg
MUFA	22.77 mg	34.4 mg
PUFA	24.22 mg	4.92 mg
SFA	47.67 mg	60.7 mg
Sodium	60.04 mg	76 mg
Potassium	274 mg	265 mg
Calcium	5.60 mg	4 mg
Magnesium	6.20 mg	12 mg
Iron	20.86 mg	5.6 mg
Zinc	94.6 mg	3.5 mg
Phosphorus	11.71 mg	334 mg
Thiamine	0.04 mg	0.14 mg
Riboflavin	0.50 mg	0.90 mg
Cobalamin	1.66 mcg	83.1 mcg

Note: SFA = Saturated Fatty Acids; PUFA = Polyunsaturated Fatty Acids; MUFA = Monounsaturated Fatty Acids. Adapted Rodrigues et al. [6]; Melo et al. [15]; Rodrigues et al. [16]; Adeyeye et al. [17].

**Table 3:** Main metabolic functions of macrominerals.

Mineral	Functions
Cácio (Ca)	Components bone mineralization, metabolic regulation, blood clotting, muscle contraction, nerve impulse transmission.
Phosphorus (P)	Components DNA and RNA, part of high-energy compounds (ATP), regulation of allosteric enzymes, component of phospholipids.
Potassium (K)	Regulation of osmotic pressure, nerve impulse transmission, regulation of acid-base balance, muscle contraction, and control of water balance.
Sulfur (S)	Sulfur amino acid component, biotin and thiamine component, mucopolysaccharides component, detoxification reactions.
Sodium (Na)	Regulation of osmotic pressure, nerve conduction, active transport of nutrients, regulation of acid-base balance, muscle contraction, and control of water balance.
Chlorine (Cl)	Regulation of osmotic pressure, regulation of acid-base balance, control of water balance, and formation of hydrochloric acid in gastric juice.
Magnesium (Mg)	Cofactor of more than 300 enzymes, components of bones, and neuromuscular activity.

Source: Adapted from Spears [58].

**Table 4:** Main metabolic functions of trace minerals.

Mineral	Function
Iron (Fe)	Oxygen transport and storage (hemoglobin, myoglobin), electron transport, enzyme component (catalase, tryptophan 5-monooxygenase, phenylalanine 4-monooxygenase, aconitase).
Zinc (Zn)	Component of more than 70 enzymes (alcohol dehydrogenase, DNA polymerase, RNA polymerase, carbonic anhydrase, carboxypeptidase, pyruvate dehydrogenase), gene expression, membrane stability.
Copper (Cu)	Component of many enzymes (lysyl oxidase, tyrosinase, cytochrome oxidase, superoxide dismutase).
Iodine (I)	Component of thyroid hormones
Manganese (Mg)	Enzyme component (pyruvate carboxylase, arginase, mitochondrial superoxide dismutase), enzyme activator (glycosyl transferases).
Cobalt (Co)	Component from vitamin B12.
Molibidênio (Mo)	Enzyme component (xanthine oxidase, sulfite oxidase, aldeide oxidase).
Selenium (Se)	Enzyme component (glutathione peroxidase, iodothyronine deiodase type I).

Source: Adapted from Spears [58].

## Data Availability

The data presented in this study are available upon reasonable request from the corresponding author.
